# Crystal structure, Hirshfeld surface analysis and inter­action energy and DFT studies of 4-[(prop-2-en-1-yl­oxy)meth­yl]-3,6-bis­(pyridin-2-yl)pyridazine

**DOI:** 10.1107/S2056989019011186

**Published:** 2019-08-20

**Authors:** Mouad Filali, Nada Kheira Sebbar, Tuncer Hökelek, Joel T. Mague, Said Chakroune, Abdessalam Ben-Tama, El Mestafa El Hadrami

**Affiliations:** a Laboratoire de Chimie Organique Appliquée, Université Sidi Mohamed Ben Abdallah, Faculté des Sciences et Techniques, Route d’Immouzzer, BP 2202, Fez, Morocco; b Laboratoire de Chimie Bioorganique Appliquée, Faculté des Sciences, Université Ibn Zohr, Agadir, Morocco; c Laboratoire de Chimie Organique Hétérocyclique URAC 21, Pôle de Compétence Pharmacochimie, Av. Ibn Battouta, BP 1014, Faculté des Sciences, Université Mohammed V, Rabat, Morocco; d Department of Physics, Hacettepe University, 06800 Beytepe, Ankara, Turkey; e Department of Chemistry, Tulane University, New Orleans, LA 70118, USA

**Keywords:** crystal structure, pyridine, pyridazine, π-stacking, DFT, Hirshfeld surface

## Abstract

The title compound, C_18_H_16_N_4_O, consists of a 3,6-bis­(pyridin-2-yl)pyridazine moiety linked to a 4-[(prop-2-en-1-yl­oxy)meth­yl] group. The pyridine-2-yl rings are oriented at a dihedral angle of 17.34 (4)° and are rotated slightly out of the plane of the pyridazine ring. In the crystal, C—H_Pyrd_⋯N_Pyrdz_ (Pyrd = pyridine and Pyrdz = pyridazine) hydrogen bonds and C—H_Prp­oxy_⋯π (Prp­oxy = prop-2-en-1-yl­oxy) inter­actions link the mol­ecules, forming deeply corrugated layers approximately parallel to the *bc* plane and stacked along the *a*-axis direction. Hirshfeld surface analysis indicates that the most important contributions for the crystal packing are from H⋯H (48.5%), H⋯C/C⋯H (26.0%) and H⋯N/N⋯H (17.1%) contacts, hydrogen bonding and van der Waals inter­actions being the dominant inter­actions in the crystal packing. Computational chemistry indicates that in the crystal, the C—H_Pyrd_⋯N_Pyrdz_ hydrogen-bond energy is 64.3 kJ mol^−1^. Density functional theory (DFT) optimized structures at the B3LYP/6–311 G(d,p) level are compared with the experimentally determined mol­ecular structure in the solid state. The HOMO–LUMO behaviour was elucidated to determine the energy gap.

## Abstract 2

The title compound consists of a 3,6-bis­(pyridin-2-yl)pyridazine unit linked to a 4-[(prop-2-en-1-yl­oxy)meth­yl] moiety. The pyridine-2-yl rings are rotated slightly out of the plane of the pyridazine ring. In the crystal, C—H⋯N hydrogen bonds and C—H⋯π inter­actions link the mol­ecules, forming deeply corrugated layers approximately parallel to the *bc* plane and stacked along the *a*-axis direction.

## Chemical context   

3,6-Di(pyridin-2-yl)pyridazine and its derivatives are aromatic heterocyclic organic compounds. The syntheses of 3,6-di(pyridin-2-yl)pyridazine and its derivatives based on polyheterocycles have attracted considerable attention from pharmacists in the last few decades as they function as important pharmacophores in medicinal chemistry and pharmacology (Filali *et al.*, 2019 ▸). 5-[3,6-Di(pyridin-2-yl)pyridazine-4-yl]-2′-de­oxy­uridine-5′-*O*-triphosphate can be used as a potential substrate for fluorescence detection and imaging of DNA (Kore *et al.*, 2015 ▸). The systems containing this moiety have also shown remarkable corrosion inhibitory (Khadiri *et al.*, 2016 ▸). Heterocyclic mol­ecules such as 3,6-bis(2′-pyrid­yl)-1,2,4,5-tetra­zine have been used in transition-metal chemistry (Kaim & Kohlmann, 1987 ▸). It is a bidentate chelate ligand popular in coordination chemistry and complexes of a wide range of metals, including iridium and palladium (Tsukada *et al.*, 2001 ▸). As a continuation of our research work devoted to the development of 3,6-di(pyridin-2-yl)pyridazine derivatives (Filali *et al.*, 2019 ▸), we report herein the synthesis and the mol­ecular and crystal structures along with the Hirshfeld surface analysis and the inter­molecular inter­action energies and density functional theory (DFT) calculations for 4-[(prop-2-en-1-yl­oxy)meth­yl]-3,6-bis­(pyridin-2-yl)pyridazine.
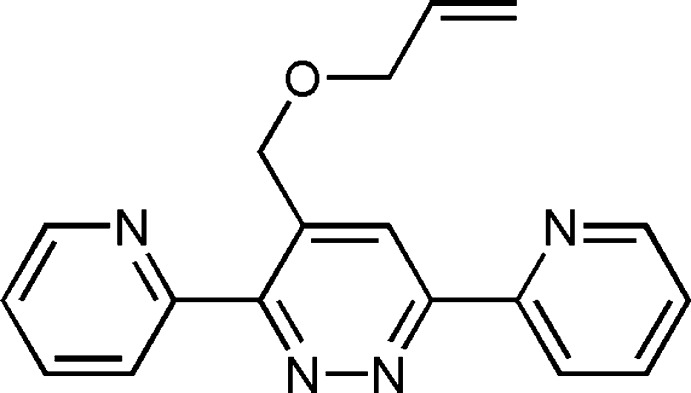


## Structural commentary   

**Figure 1:**
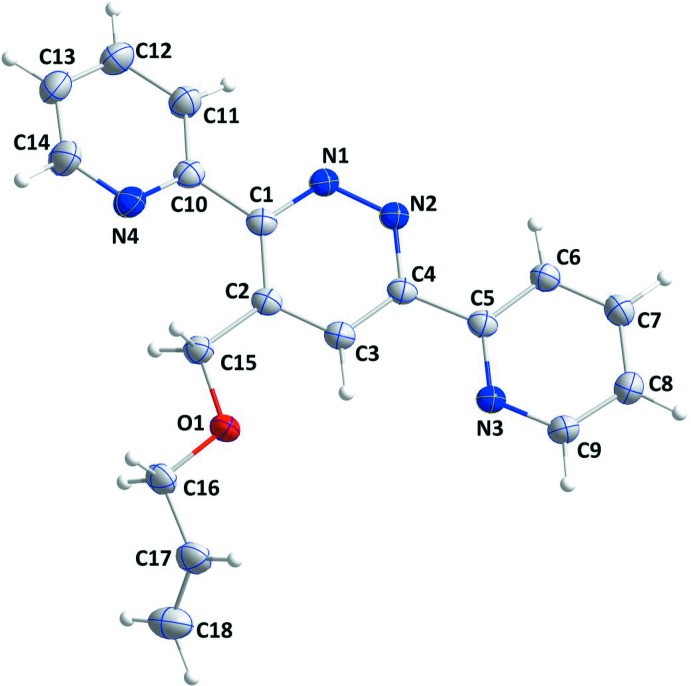
The mol­ecular structure of the title compound with the atom-numbering scheme. Displacement ellipsoids are drawn at the 50% probability level.

The title mol­ecule contains two pyridine and one pyradizine rings (Fig. 1 ▸). The pyradizine ring of the 3,6-bis­(pyridin-2-yl)pyridazine unit is linked to the 4-[(prop-2-en-1-yl­oxy)meth­yl] moiety (Fig. 1 ▸). Pyridazine ring *A* (N1/N2/C1–C4) is oriented at dihedral angles of 2.64 (3) and 15.06 (4)°, respectively, to the pyridine rings *B* (N3/C5–C9) and *C* (N4/C10–C14), while the dihedral angle between the two pyridine rings is 17.34 (4)°. Atom C15 is at a distance of 0.0405 (12) Å from the best plane of pyridazine ring. The 4-[(prop-2-en-1-yl­oxy)meth­yl] moiety is nearly co-planar with the pyradizine ring, as indicated by the O1—C15—C2—C3 torsion angle of −2.59 (14)°.

## Supra­molecular features   

**Figure 2:**
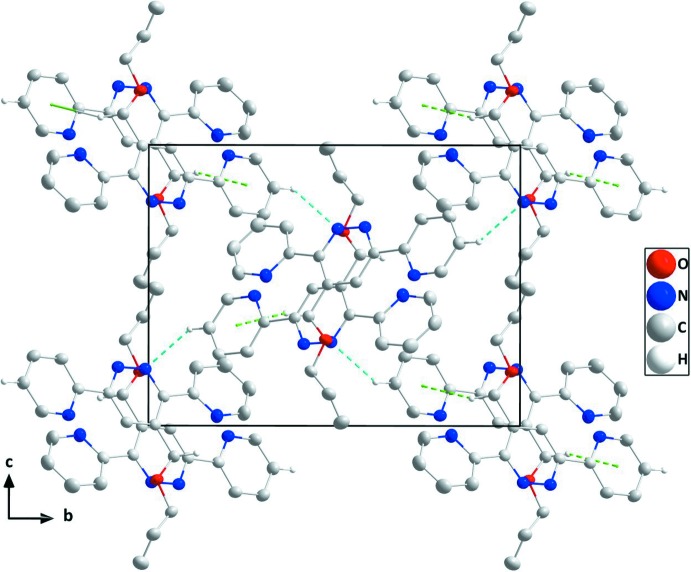
A partial packing diagram viewed along the *a*-axis direction with C—H_Pyrd_⋯N_Pyrdz_ hydrogen bonds and C—H_Prp­oxy_⋯π inter­actions shown, respectively, as light blue and green dashed lines.

In the crystal, C—H_Pyrd_⋯N_Pyrdz_ (Pyrd = pyridine, Pyrdz = pyridazine) hydrogen bonds and C—H_Prp­oxy_⋯*Cg* ^i^ [symmetry code: (i) 1 − *x*, 1 − *y*, 1 - *z; *Cg** is the centroid of pyridine ring *B* (N3/C5–C9); Prp­oxy = prop-2-en-1-yl­oxy] (Table 1 ▸) inter­actions link the mol­ecules, forming deeply corrugated layers approximately parallel to the *bc* plane and stacked along the *a*-axis direction (Figs. 2 ▸ and 3 ▸).

## Hirshfeld surface analysis   

**Figure 4:**
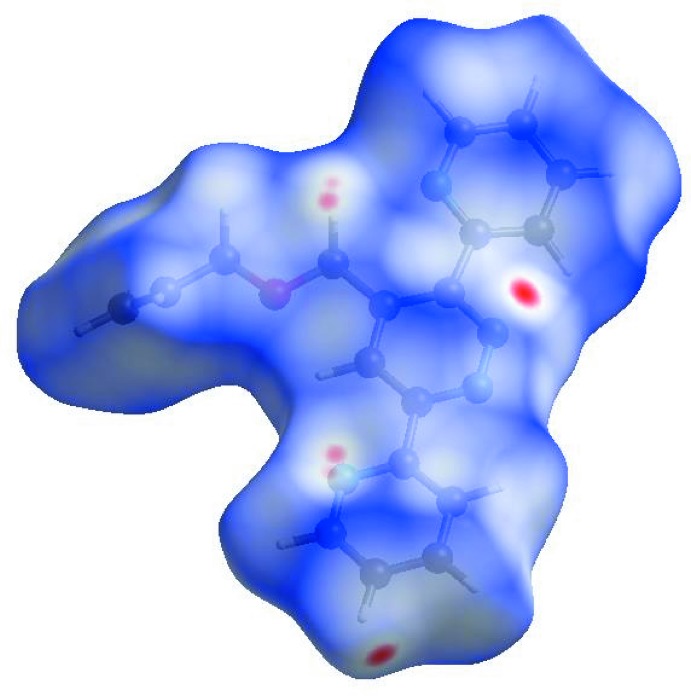
View of the three-dimensional Hirshfeld surface of the title compound plotted over *d*_norm_ in the range −0.1063 to 1.1444 a.u.

**Figure 5:**
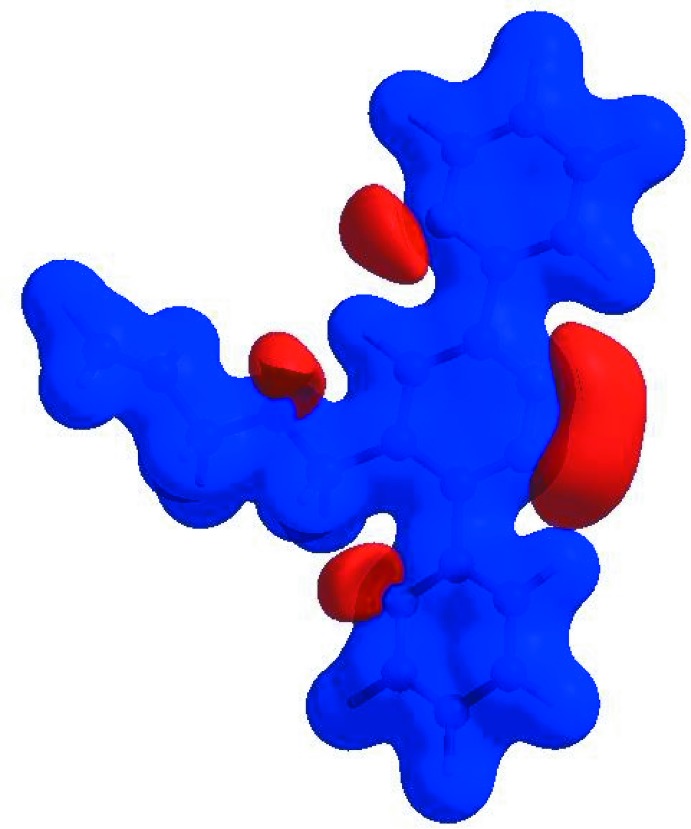
View of the three-dimensional Hirshfeld surface of the title compound plotted over electrostatic potential energy in the range −0.0500 to 0.0500 a.u. using the STO-3 G basis set at the Hartree–Fock level of theory. Hydrogen-bond donors and acceptors are shown as blue and red regions, respectively, around the atoms, corresponding to positive and negative potentials.

**Figure 6:**
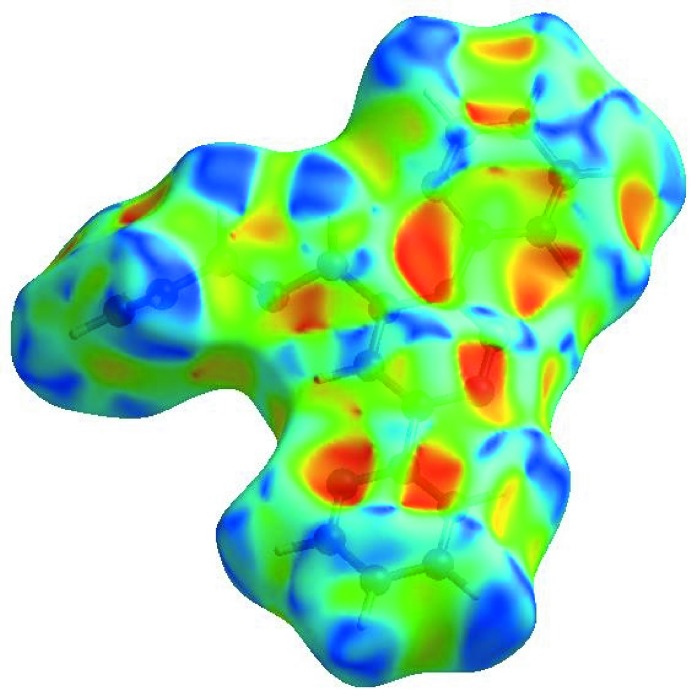
Hirshfeld surface of the title compound plotted over shape-index.

In order to visualize the inter­molecular inter­actions, a Hirshfeld surface (HS) analysis (Hirshfeld, 1977 ▸; Spackman & Jayatilaka, 2009 ▸) was carried out by using *CrystalExplorer17.5* (Turner *et al.*, 2017 ▸). In the HS plotted over *d*_norm_ (Fig. 4 ▸), white areas indicate contacts with distances equal to the sum of van der Waals radii, and red and blue areas indicate distances shorter (in close contact) or longer (distinct contact) than the van der Waals radii (Venkatesan *et al.*, 2016 ▸). The bright-red spots appearing near N1 and hydrogen atoms H8 and H15*B* indicate their roles as donors and/or acceptors; they also appear as blue and red regions corresponding to positive and negative potentials on the HS mapped over electrostatic potential (Spackman *et al.*, 2008 ▸; Jayatilaka *et al.*, 2005 ▸) shown in Fig. 5 ▸. The blue regions indicate positive electrostatic potential (hydrogen-bond donors), while the red regions indicate negative electrostatic potential (hydrogen-bond acceptors). The shape-index of the HS is a tool to visualize π–π stacking by the presence of adjacent red and blue triangles; if there are no adjacent red and/or blue triangles, then there are no π–π inter­actions. Fig. 6 ▸ clearly suggest that there are no π–π inter­actions in (I).

**Figure 7:**
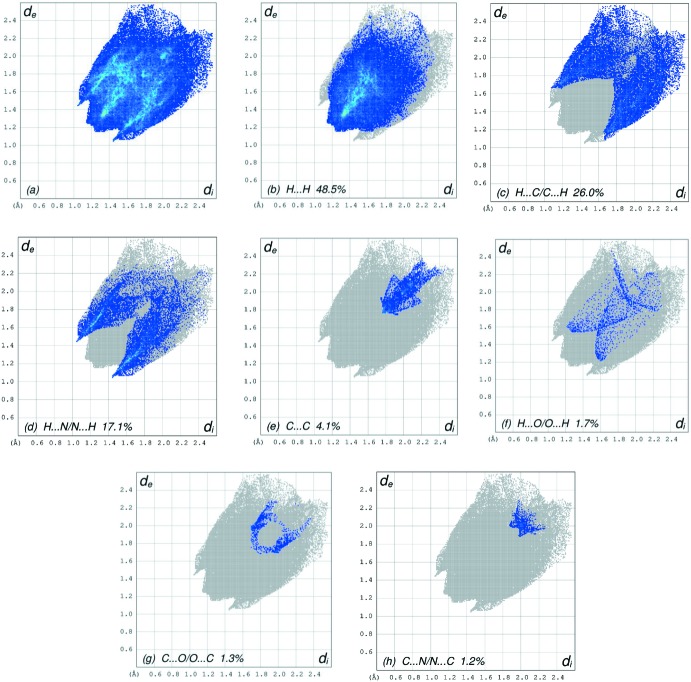
The full two-dimensional fingerprint plots for the title compound, showing (*a*) all inter­actions, and delineated into (*b*) H⋯H, (*c*) H⋯C/C⋯H, (*d*) H⋯N/N⋯H, (*e*) C⋯C, (*f*) H⋯O/O⋯H, (*g*) C⋯O/O⋯C and (*h*) C ⋯ N/N⋯C inter­actions. The *d*_i_ and *d*_e_ values are the closest inter­nal and external distances (in Å) from given points on the Hirshfeld surface.

The overall two-dimensional fingerprint plot, Fig. 7 ▸ *a*, and those delineated into H ⋯ H, H⋯C/C⋯H, H⋯N/N⋯H, C⋯C, H⋯O/O⋯H, O⋯C/C ⋯ O and C⋯N/N⋯C contacts (McKinnon *et al.*, 2007 ▸) are illustrated in Fig. 7 ▸ *b*–*h*, respectively, together with their relative contributions to the Hirshfeld surface. The most important inter­action is H⋯H (Table 2 ▸), contributing 48.5% to the overall crystal packing, which is reflected in Fig. 7 ▸ *b* as widely scattered points of high density, due to the large hydrogen content of the mol­ecule, with the tips at *d*_e_ + *d*_i_ ∼2.39 Å. In the presence of C—H⋯π inter­actions, the pair of characteristic wings in the fingerprint plot delineated into H⋯C/C⋯H contacts (26.0% contribution), Fig. 7 ▸ *c*, has a pair of spikes with the tips at d_e_ + d_i_ = 2.72 Å. The pair of the scattered points of wings in the fingerprint plots delineated into H⋯N/N⋯H (17.1% contribution), Fig. 7 ▸ *d*, has a symmetrical distribution of points with the edges at *d*_e_ + *d*_i_ = 2.50 Å. The C⋯C contacts, Fig. 7 ▸ *e*, have an arrow-shaped distribution of points with the tip at *d*_e_ = *d*_i_ = 1.76 Å. The pair of characteristic wings in the fingerprint plot delineated into H⋯O/O⋯H contacts (1.7% contribution) Fig. 7 ▸ *f*, has a pair of spikes with the tips at *d*_e_ + *d*_i_ = 2.82 Å. Finally, in the fingerprint plots delineated into C⋯O/O⋯C (1.3%) and C⋯N/N⋯C (1.2%) contacts, Fig. 7 ▸ *g* and Fig. 7 ▸ *h*, the tips are at *d*_e_ = *d*_i_ = 1.65 Å and 3.87 Å, respectively.

**Figure 8:**
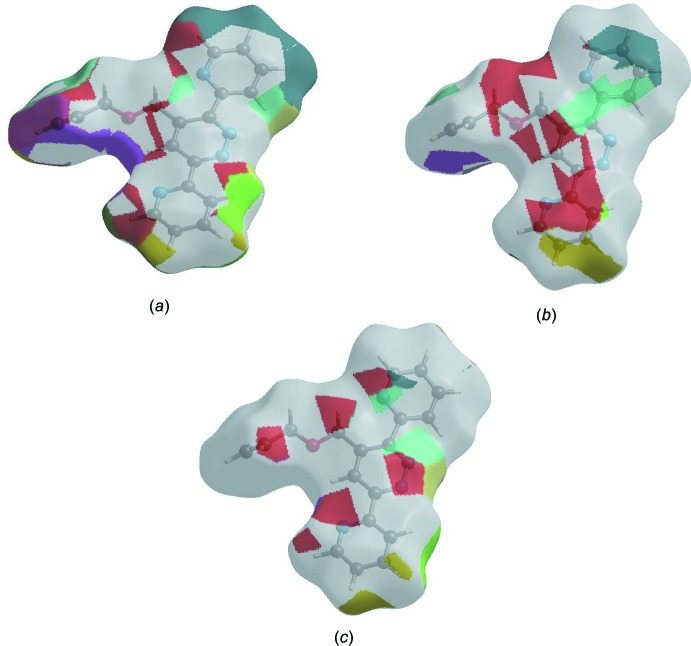
The Hirshfeld surface representations with the function *d*_norm_ plotted onto the surface for (*a*) H⋯H, (*b*) H⋯C/C⋯H and (*c*) H⋯N/N⋯H inter­actions.

The Hirshfeld surface representations with the function *d*_norm_ plotted onto the surface are shown for the H⋯H, H⋯C/C⋯H and H⋯N/N⋯H inter­actions in Fig. 8 ▸ *a-*-*c*, respectively.

The Hirshfeld surface analysis confirms the importance of H-atom contacts in establishing the packing. The large number of H⋯H, H⋯C/C⋯H and H ⋯ N/N⋯H inter­actions suggest that van der Waals inter­actions and hydrogen bonding play the major roles in the crystal packing (Hathwar *et al.*, 2015 ▸).

## Inter­action energy calculations   

The inter­molecular inter­action energies were calculated using the CE–B3LYP/6–31G(d,p) energy model available in *CrystalExplorer17.5* (Turner *et al.*, 2017 ▸), where a cluster of mol­ecules would need to be generated by applying crystallographic symmetry operations with respect to a selected central mol­ecule within the radius of 3.8 Å by default (Turner *et al.*, 2014 ▸). The total inter­molecular energy (*E*_tot_) is the sum of electrostatic (*E*_ele_), polarization (*E*_pol_), dispersion (*E*_dis_) and exchange-repulsion (*E*_rep_) energies (Turner *et al.*, 2015 ▸) with scale factors of 1.057, 0.740, 0.871 and 0.618, respectively (Mackenzie *et al.*, 2017 ▸). The hydrogen-bonding inter­action energy (in kJ mol^−1^) was calculated as −15.0 (*E*_ele_), −3.2 (*E*_pol_), −81.9 (*E*_dis_), 40.9 (*E*_rep_) and −64.3 (*E*_tot_) for the C—H_Pyrd_⋯N_Pyrdz_ hydrogen bond.

## DFT calculations   

**Figure 9:**
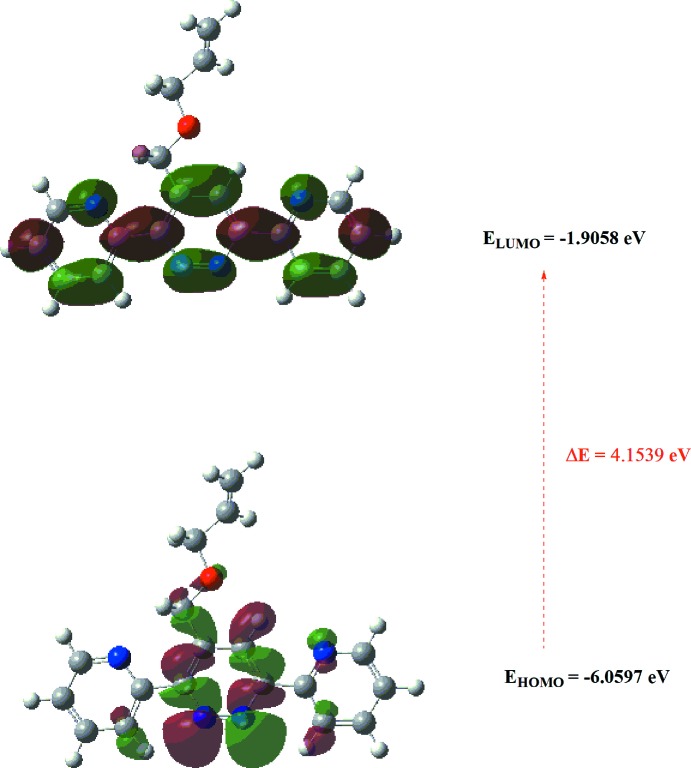
The energy band gap of the title compound.

The optimized structure of the title compound in the gas phase was generated theoretically *via* density functional theory (DFT) using standard B3LYP functional and 6–311 G(d,p) basis-set calculations (Becke, 1993 ▸) as implemented in *GAUSSIAN 09* (Frisch *et al.*, 2009 ▸). The theoretical and experimental results were in good agreement (Table 3 ▸). The highest-occupied mol­ecular orbital (HOMO), acting as an electron donor, and the lowest-unoccupied mol­ecular orbital (LUMO), acting as an electron acceptor, are very important parameters for quantum chemistry. When the energy gap is small, the mol­ecule is highly polarizable and has high chemical reactivity. The DFT calculations provide some important information on the reactivity and site selectivity of the mol­ecular framework. *E*_HOMO_ and *E*_LUMO_ clarify the inevitable charge-exchange collaboration inside the studied material, and are given in Table 4 ▸ along with the electronegativity (χ), hardness (η), potential (μ), electrophilicity (ω) and softness (*σ*). The significance of η and *σ* is to evaluate both the reactivity and stability. The electron transition from the HOMO to the LUMO energy level is shown in Fig. 9 ▸. The HOMO and LUMO are localized in the plane extending from the whole 4-[(prop-2-en-1-yl­oxy)meth­yl]-3,6-bis­(pyridin-2-yl)pyridazine ring. The energy band gap [Δ*E* = *E*_LUMO_ − *E*_HOMO_] of the mol­ecule is 4.1539 eV, and the frontier mol­ecular orbital energies, *E*_HOMO_ and *E*_LUMO_ are −6.0597 and −1.9058 eV, respectively.

## Database survey   

Silver(I) complexes supported by 3,6-di(pyridin-2-yl)pyridazine ligands have been reported (Constable *et al.*, 2008 ▸). Three other metal complexes including 3,6-di(pyridin-2-yl)pyridazine have also been reported, namely aqua­bis­[3,6-bis­(pyridin-2-yl)pyridazine-κ^2^ *N* ^1^,*N* ^6^]copper(II) bis­(tri­fluoro­methane­sulfonate) (Showrilu *et al.*, 2017 ▸), tetra­kis­[μ-3,6-di(pyridin-2-yl)pyridazine]bis­(μ-hydroxo)bis­(μ-aqua)­tetra­nickel(II) hexa­kis­(nitrate) tetra­deca­hydrate (Marino *et al.*, 2019 ▸) and *catena*-[[μ_2_-3,6-di(pyridin-2-yl)pyridazine]bis­(μ_2_-azido)­dizaidodicopper monohydrate] (Mastropietro *et al.*, 2013 ▸).

## Synthesis and crystallization   

THF (20 ml), [3,6-di(pyridin-2-yl)pyridazin-4-yl]methanol (3 mmol), 1.8 eq. of NaH and 0.04 eq. of 18-crown ether A were added to a conical flask and stirred for 10 min at room temperature. Then 1.2 eq of propargyl allyl chloride was added to the reaction mixture and stirred for 48 h. The solvent was then evaporated off and the required organic compound was obtained by liquid–liquid extraction using di­chloro­methane. The organic phase was dried with sodium sulfate (Na_2_SO_4_), and then evaporated. The product obtained was separated by chromatography on a column of silica gel. The isolated solid was recrystallized from hexane-di­chloro­methane (1:1) to afford colourless crystals (yield: 87%, m.p. 376 K).

## Refinement   

Crystal data, data collection and structure refinement details are summarized in Table 5 ▸. The hydrogen atoms were located in a difference-Fourier map and refined freely.

## Supplementary Material

Crystal structure: contains datablock(s) I, global. DOI: 10.1107/S2056989019011186/lh5915sup1.cif

Structure factors: contains datablock(s) I. DOI: 10.1107/S2056989019011186/lh5915Isup2.hkl

Supporting information file. DOI: 10.1107/S2056989019011186/lh5915Isup3.cdx

Supporting information file. DOI: 10.1107/S2056989019011186/lh5915Isup4.cml

CCDC reference: 1946685

Additional supporting information:  crystallographic information; 3D view; checkCIF report

## Figures and Tables

**Figure 3:**
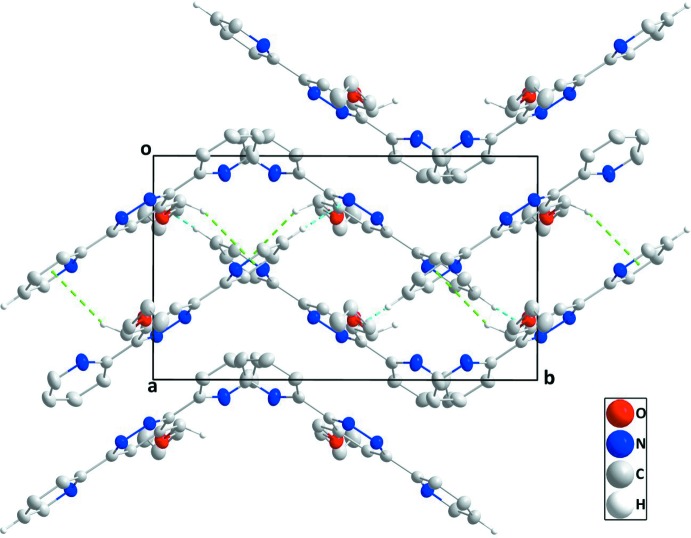
A partial packing diagram viewed along the *c*-axis direction with C—H_Pyrd_⋯N_Pyrdz_ hydrogen bonds and C—H_Prp­oxy_⋯π inter­actions shown, respectively, as light-blue and green dashed lines.

**Table 1:** Hydrogen-bond geometry (Å, °) *Cg* is the centroid of pyridine ring *B* (N3/C5—C9).

*D*—H⋯*A*	*D*—H	H⋯*A*	*D*⋯*A*	*D*—H⋯*A*
C8—H8⋯N1^vi^	0.966 (16)	2.585 (16)	3.4104 (15)	143.4 (12)
C15—H15*B*⋯*Cg* ^v^	0.994 (15)	2.990 (15)	3.8760 (13)	149.0 (11)

Symmetry codes: (v) 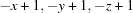; (vi) 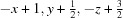.

**Table 2:** Selected interatomic distances (Å)

O1⋯C11^i^	3.2992 (16)	C3⋯C11^i^	3.5866 (17)
O1⋯H3	2.232 (14)	C6⋯C12^iv^	3.5808 (18)
O1⋯H11^i^	2.850 (16)	C8⋯C10^vi^	3.5797 (17)
N1⋯C8^ii^	3.4105 (15)	C11⋯C15^i^	3.5633 (18)
N4⋯C15	2.7895 (16)	C1⋯H7^ii^	2.925 (17)
N1⋯H8^ii^	2.586 (15)	C6⋯H16*B* ^v^	2.933 (15)
N1⋯H11	2.441 (16)	C9⋯H15*B* ^v^	2.842 (15)
N1⋯H15*A* ^i^	2.713 (14)	C18⋯H8^vii^	2.920 (16)
N2⋯H18*B* ^iii^	2.86 (2)	H6⋯H9^viii^	2.56 (2)
N2⋯H13^iv^	2.744 (17)	H8⋯N1^vi^	2.586 (16)
N2⋯H6	2.455 (15)	H11⋯H16*A* ^i^	2.57 (2)
N3⋯H3	2.522 (14)	H12⋯C6^ix^	2.886 (18)
N3⋯H15*B* ^v^	2.652 (15)	H12⋯H14^x^	2.53 (3)
N4⋯H15*A*	2.632 (14)	H13⋯H18*B* ^xi^	2.55 (3)
N4⋯H15*B*	2.485 (14)	H15*A*⋯H16*A*	2.36 (2)
C1⋯C7^ii^	3.5853 (17)	H15*B*⋯H16*B*	2.38 (2)
C2⋯C10^i^	3.5420 (15)	H16*A*⋯H18*A*	2.33 (2)

Symmetry codes: (i) 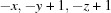; (ii) 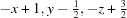; (iii) 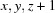; (iv) 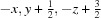; (v) 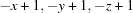; (vi) 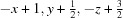; (vii) 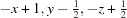; (viii) 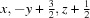; (ix) 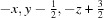; (x) 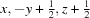; (xi) 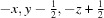.

**Table 3:** Comparison of the selected (X-ray and DFT) geometric data (Å, °)

Bonds/angles	X-ray	B3LYP/6–311G(d,p)
O1—C15	1.4224 (13)	1.45001
O1—C16	1.4237 (14)	1.45647
N1—N2	1.3322 (13)	1.33754
N1—C1	1.3434 (15)	1.36030
N2—C4	1.3386 (14)	1.35694
N3—C9	1.3370 (15)	1.34713
N3—C5	1.3445 (14)	1.35667
N4—C10	1.3362 (15)	1.35644
N4—C14	1.3400 (17)	1.34940
C15—O1—C16	111.09 (9)	112.34477
N2—N1—C1	121.37 (9)	121.70569
N1—N2—C4	119.14 (9)	119.30129
C9—N3—C5	117.07 (10)	118.58051
C10—N4—C14	117.42 (11)	119.00361
N1—C1—C2	121.82 (10)	121.25910
N1—C1—C10	113.24 (10)	113.37034
N2—C4—C3	122.25 (10)	121.78580
N2—C4—C5	115.80 (10)	116.28262
C3—C4—C5	121.95 (10)	121.93158
N3—C5—C6	122.61 (10)	122.07926
N3—C5—C4	116.15 (10)	116.59443

**Table 4:** Calculated energies for the title compound

Total energy, *TE* (eV)	−26922.3681
*E*_HOMO_ (eV)	−6.0597
*E*_LUMO_ (eV)	−1.9058
Energy gap, *ΔE* (eV)	4.1539
Dipole moment *μ* (Debye)	1.6276
Ionization potential, *I* (eV)	6.0597
Electron affinity, *A*	1.9058
Electronegativity, *χ*	3.9827
Hardness, *η*	2.0769
Electrophilicity index, *ω*	3.8186
Softness, *σ*	0.4815
Fraction of electrons transferred, *ΔN*	0.7264

**Table 5:** Experimental details

Crystal data	
Chemical formula	C_18_H_16_N_4_O
*M*_r_	304.35
Crystal system, space group	Monoclinic, *P*2_1_/*c*
Temperature (K)	150
*a*, *b*, *c* (Å)	8.9420 (2), 15.1130 (3), 11.5829 (3)
β (°)	100.132 (1)
*V* (Å^3^)	1540.91 (6)
*Z*	4
Radiation type	Cu *K*α
μ (mm^−1^)	0.68
Crystal size (mm)	0.26 × 0.24 × 0.08

Data collection	
Diffractometer	Bruker D8 VENTURE PHOTON 100 CMOS
Absorption correction	Multi-scan (*SADABS*; Krause *et al.*, 2015 ▸)
*T*_min_, *T*_max_	0.86, 0.95
No. of measured, independent and observed [*I* > 2σ(*I*)] reflections	11678, 3051, 2688
*R*_int_	0.029
(sin θ/λ)_max_ (Å^−1^)	0.625

Refinement	
*R*[*F* ^2^ > 2σ(*F* ^2^)], *wR*(*F* ^2^), *S*	0.037, 0.101, 1.04
No. of reflections	3051
No. of parameters	273
H-atom treatment	All H-atom parameters refined
Δρ_max_, Δρ_min_ (e Å^−3^)	0.18, −0.15

Computer programs: *APEX3* and *SAINT* (Bruker, 2016 ▸), *SAINT* (Bruker, 2016 ▸), *SHELXT* (Sheldrick, 2015*a* ▸), *SHELXL2018* (Sheldrick, 2015*b* ▸), *DIAMOND* (Brandenburg & Putz, 2012 ▸) and *SHELXTL* (Sheldrick, 2008 ▸).

## supplementary crystallographic information

### Crystal data

**Table:**

C_18_H_16_N_4_O	*F*(000) = 640
*M**_r_* = 304.35	*D*_x_ = 1.312 Mg m^−^^3^
Monoclinic, *P*2_1_/*c*	Cu *K*α radiation, λ = 1.54178 Å
*a* = 8.9420 (2) Å	Cell parameters from 9267 reflections
*b* = 15.1130 (3) Å	θ = 4.9–74.5°
*c* = 11.5829 (3) Å	µ = 0.68 mm^−^^1^
β = 100.132 (1)°	*T* = 150 K
*V* = 1540.91 (6) Å^3^	Plate, colourless
*Z* = 4	0.26 × 0.24 × 0.08 mm

### Data collection

**Table:**

Bruker D8 VENTURE PHOTON 100 CMOS diffractometer	3051 independent reflections
Radiation source: INCOATEC IµS micro-focus source	2688 reflections with *I* > 2σ(*I*)
Mirror monochromator	*R*_int_ = 0.029
Detector resolution: 10.4167 pixels mm^-1^	θ_max_ = 74.5°, θ_min_ = 4.9°
ω scans	*h* = −10→10
Absorption correction: multi-scan (*SADABS*; Krause *et al.*, 2015)	*k* = −18→18
*T*_min_ = 0.86, *T*_max_ = 0.95	*l* = −14→13
11678 measured reflections	

### Refinement

**Table:**

Refinement on *F*^2^	Secondary atom site location: difference Fourier map
Least-squares matrix: full	Hydrogen site location: difference Fourier map
*R*[*F*^2^ > 2σ(*F*^2^)] = 0.037	All H-atom parameters refined
*wR*(*F*^2^) = 0.101	*w* = 1/[σ^2^(*F*_o_^2^) + (0.0534*P*)^2^ + 0.3637*P*] where *P* = (*F*_o_^2^ + 2*F*_c_^2^)/3
*S* = 1.04	(Δ/σ)_max_ < 0.001
3051 reflections	Δρ_max_ = 0.18 e Å^−^^3^
273 parameters	Δρ_min_ = −0.15 e Å^−^^3^
0 restraints	Extinction correction: *SHELXL2018* (Sheldrick, 2015*b*), Fc^*^=kFc[1+0.001xFc^2^λ^3^/sin(2θ)]^-1/4^
Primary atom site location: dual space	Extinction coefficient: 0.0046 (5)

### Special details

**Table:**

Geometry. All esds (except the esd in the dihedral angle between two l.s. planes) are estimated using the full covariance matrix. The cell esds are taken into account individually in the estimation of esds in distances, angles and torsion angles; correlations between esds in cell parameters are only used when they are defined by crystal symmetry. An approximate (isotropic) treatment of cell esds is used for estimating esds involving l.s. planes.
Refinement. Refinement of F^2^ against ALL reflections. The weighted R-factor wR and goodness of fit S are based on F^2^, conventional R-factors R are based on F, with F set to zero for negative F^2^. The threshold expression of F^2^ > 2sigma(F^2^) is used only for calculating R-factors(gt) etc. and is not relevant to the choice of reflections for refinement. R-factors based on F^2^ are statistically about twice as large as those based on F, and R- factors based on ALL data will be even larger.

### Fractional atomic coordinates and isotropic or equivalent isotropic displacement parameters (Å^2^)

**Table:**

	*x*	*y*	*z*	*U*_iso_*/*U*_eq_	
O1	0.27225 (10)	0.47749 (5)	0.30640 (7)	0.0319 (2)	
N1	0.19512 (11)	0.50876 (6)	0.70191 (8)	0.0283 (2)	
N2	0.27872 (11)	0.58215 (6)	0.70850 (8)	0.0283 (2)	
N3	0.49183 (11)	0.71463 (6)	0.53716 (8)	0.0299 (2)	
N4	0.07708 (13)	0.31558 (7)	0.53457 (10)	0.0370 (3)	
C1	0.17076 (12)	0.45900 (7)	0.60429 (10)	0.0249 (2)	
C2	0.23110 (12)	0.48225 (7)	0.50338 (10)	0.0244 (2)	
C3	0.31696 (12)	0.55846 (7)	0.51158 (10)	0.0257 (2)	
H3	0.3616 (15)	0.5785 (9)	0.4416 (12)	0.030 (3)*	
C4	0.33924 (12)	0.60672 (7)	0.61566 (9)	0.0244 (2)	
C5	0.43278 (12)	0.68871 (7)	0.63070 (10)	0.0250 (2)	
C6	0.45861 (13)	0.73441 (8)	0.73683 (10)	0.0292 (3)	
H6	0.4125 (17)	0.7131 (10)	0.8002 (13)	0.040 (4)*	
C7	0.54897 (14)	0.80913 (8)	0.74716 (11)	0.0329 (3)	
H7	0.5668 (18)	0.8438 (11)	0.8229 (15)	0.049 (4)*	
C8	0.61156 (14)	0.83614 (8)	0.65191 (11)	0.0326 (3)	
H8	0.6740 (17)	0.8887 (10)	0.6564 (14)	0.042 (4)*	
C9	0.57918 (14)	0.78700 (8)	0.54977 (11)	0.0323 (3)	
H9	0.6207 (17)	0.8041 (10)	0.4814 (14)	0.043 (4)*	
C10	0.07702 (12)	0.37891 (7)	0.61505 (10)	0.0269 (3)	
C11	−0.00417 (14)	0.37100 (9)	0.70670 (11)	0.0346 (3)	
H11	−0.0048 (18)	0.4190 (11)	0.7619 (14)	0.044 (4)*	
C12	−0.08744 (15)	0.29497 (9)	0.71477 (12)	0.0404 (3)	
H12	−0.147 (2)	0.2897 (11)	0.7774 (16)	0.058 (5)*	
C13	−0.08794 (16)	0.22904 (9)	0.63254 (12)	0.0405 (3)	
H13	−0.1415 (19)	0.1766 (12)	0.6368 (14)	0.050 (4)*	
C14	−0.00414 (18)	0.24243 (9)	0.54512 (13)	0.0440 (3)	
H14	−0.0035 (19)	0.1969 (11)	0.4852 (15)	0.052 (5)*	
C15	0.20351 (13)	0.43087 (8)	0.39007 (10)	0.0279 (3)	
H15A	0.0898 (16)	0.4252 (9)	0.3600 (12)	0.032 (3)*	
H15B	0.2466 (16)	0.3702 (10)	0.4016 (12)	0.036 (4)*	
C16	0.23624 (15)	0.43750 (8)	0.19364 (10)	0.0321 (3)	
H16A	0.1233 (17)	0.4420 (9)	0.1637 (13)	0.037 (4)*	
H16B	0.2670 (16)	0.3739 (10)	0.1992 (13)	0.038 (4)*	
C17	0.32018 (17)	0.48569 (9)	0.11329 (11)	0.0377 (3)	
H17	0.436 (2)	0.4884 (12)	0.1409 (17)	0.065 (5)*	
C18	0.2574 (2)	0.51972 (11)	0.01194 (13)	0.0497 (4)	
H18A	0.150 (2)	0.5087 (12)	−0.0128 (17)	0.065 (6)*	
H18B	0.314 (2)	0.5515 (13)	−0.0442 (18)	0.068 (5)*	

### Atomic displacement parameters (Å^2^)

**Table:**

	*U*^11^	*U*^22^	*U*^33^	*U*^12^	*U*^13^	*U*^23^
O1	0.0418 (5)	0.0330 (4)	0.0225 (4)	−0.0060 (3)	0.0099 (3)	−0.0033 (3)
N1	0.0316 (5)	0.0278 (5)	0.0259 (5)	−0.0019 (4)	0.0065 (4)	0.0011 (4)
N2	0.0321 (5)	0.0283 (5)	0.0251 (5)	−0.0025 (4)	0.0070 (4)	0.0001 (4)
N3	0.0351 (5)	0.0306 (5)	0.0249 (5)	−0.0051 (4)	0.0079 (4)	−0.0004 (4)
N4	0.0503 (7)	0.0280 (5)	0.0348 (6)	−0.0061 (4)	0.0129 (5)	−0.0010 (4)
C1	0.0243 (5)	0.0250 (5)	0.0252 (6)	0.0039 (4)	0.0035 (4)	0.0027 (4)
C2	0.0249 (5)	0.0246 (5)	0.0236 (6)	0.0045 (4)	0.0034 (4)	0.0014 (4)
C3	0.0272 (5)	0.0272 (5)	0.0230 (6)	0.0019 (4)	0.0051 (4)	0.0013 (4)
C4	0.0244 (5)	0.0259 (5)	0.0229 (5)	0.0027 (4)	0.0038 (4)	0.0020 (4)
C5	0.0243 (5)	0.0270 (5)	0.0235 (6)	0.0023 (4)	0.0034 (4)	0.0015 (4)
C6	0.0302 (6)	0.0339 (6)	0.0239 (6)	−0.0026 (5)	0.0059 (4)	−0.0009 (4)
C7	0.0361 (6)	0.0348 (6)	0.0274 (6)	−0.0041 (5)	0.0044 (5)	−0.0053 (5)
C8	0.0350 (6)	0.0297 (6)	0.0325 (7)	−0.0060 (5)	0.0047 (5)	−0.0006 (5)
C9	0.0381 (6)	0.0325 (6)	0.0277 (6)	−0.0065 (5)	0.0089 (5)	0.0015 (5)
C10	0.0264 (5)	0.0262 (5)	0.0274 (6)	0.0024 (4)	0.0026 (4)	0.0042 (4)
C11	0.0336 (6)	0.0361 (6)	0.0355 (7)	−0.0049 (5)	0.0101 (5)	−0.0016 (5)
C12	0.0378 (7)	0.0455 (7)	0.0399 (7)	−0.0096 (6)	0.0121 (6)	0.0032 (6)
C13	0.0434 (7)	0.0338 (7)	0.0437 (8)	−0.0110 (6)	0.0059 (6)	0.0055 (5)
C14	0.0603 (9)	0.0310 (6)	0.0424 (8)	−0.0107 (6)	0.0140 (6)	−0.0031 (6)
C15	0.0320 (6)	0.0270 (6)	0.0257 (6)	−0.0007 (4)	0.0079 (5)	−0.0004 (4)
C16	0.0412 (7)	0.0299 (6)	0.0254 (6)	0.0005 (5)	0.0066 (5)	−0.0052 (4)
C17	0.0486 (8)	0.0373 (7)	0.0293 (7)	0.0008 (6)	0.0131 (6)	−0.0040 (5)
C18	0.0689 (11)	0.0476 (8)	0.0355 (8)	0.0083 (7)	0.0170 (7)	0.0059 (6)

### Geometric parameters (Å, º)

**Table:**

O1—C15	1.4224 (13)	C8—C9	1.3836 (17)
O1—C16	1.4237 (14)	C8—H8	0.966 (16)
N1—N2	1.3322 (13)	C9—H9	0.968 (15)
N1—C1	1.3434 (15)	C10—C11	1.3926 (17)
N2—C4	1.3386 (14)	C11—C12	1.3813 (18)
N3—C9	1.3370 (15)	C11—H11	0.968 (17)
N3—C5	1.3445 (14)	C12—C13	1.378 (2)
N4—C10	1.3362 (15)	C12—H12	0.974 (17)
N4—C14	1.3400 (17)	C13—C14	1.377 (2)
C1—C2	1.4151 (15)	C13—H13	0.931 (18)
C1—C10	1.4901 (15)	C14—H14	0.978 (17)
C2—C3	1.3782 (15)	C15—H15A	1.019 (14)
C2—C15	1.5075 (15)	C15—H15B	0.994 (15)
C3—C4	1.3930 (15)	C16—C17	1.4851 (18)
C3—H3	1.012 (14)	C16—H16A	1.011 (15)
C4—C5	1.4880 (15)	C16—H16B	0.999 (15)
C5—C6	1.3934 (16)	C17—C18	1.314 (2)
C6—C7	1.3813 (17)	C17—H17	1.03 (2)
C6—H6	0.959 (15)	C18—H18A	0.96 (2)
C7—C8	1.3836 (17)	C18—H18B	1.01 (2)
C7—H7	1.010 (17)		
			
O1···C11^i^	3.2992 (16)	C3···C11^i^	3.5866 (17)
O1···H3	2.232 (14)	C6···C12^iv^	3.5808 (18)
O1···H11^i^	2.850 (16)	C8···C10^vi^	3.5797 (17)
N1···C8^ii^	3.4105 (15)	C11···C15^i^	3.5633 (18)
N4···C15	2.7895 (16)	C1···H7^ii^	2.925 (17)
N1···H8^ii^	2.586 (15)	C6···H16B^v^	2.933 (15)
N1···H11	2.441 (16)	C9···H15B^v^	2.842 (15)
N1···H15A^i^	2.713 (14)	C18···H8^vii^	2.920 (16)
N2···H18B^iii^	2.86 (2)	H6···H9^viii^	2.56 (2)
N2···H13^iv^	2.744 (17)	H8···N1^vi^	2.586 (16)
N2···H6	2.455 (15)	H11···H16A^i^	2.57 (2)
N3···H3	2.522 (14)	H12···C6^ix^	2.886 (18)
N3···H15B^v^	2.652 (15)	H12···H14^x^	2.53 (3)
N4···H15A	2.632 (14)	H13···H18B^xi^	2.55 (3)
N4···H15B	2.485 (14)	H15A···H16A	2.36 (2)
C1···C7^ii^	3.5853 (17)	H15B···H16B	2.38 (2)
C2···C10^i^	3.5420 (15)	H16A···H18A	2.33 (2)
			
C15—O1—C16	111.09 (9)	N4—C10—C1	117.02 (10)
N2—N1—C1	121.37 (9)	C11—C10—C1	120.65 (10)
N1—N2—C4	119.14 (9)	C12—C11—C10	118.85 (12)
C9—N3—C5	117.07 (10)	C12—C11—H11	120.9 (9)
C10—N4—C14	117.42 (11)	C10—C11—H11	120.3 (10)
N1—C1—C2	121.82 (10)	C13—C12—C11	119.41 (12)
N1—C1—C10	113.24 (10)	C13—C12—H12	121.3 (10)
C2—C1—C10	124.93 (10)	C11—C12—H12	119.3 (10)
C3—C2—C1	116.06 (10)	C14—C13—C12	117.79 (12)
C3—C2—C15	119.63 (10)	C14—C13—H13	121.0 (10)
C1—C2—C15	124.29 (10)	C12—C13—H13	121.2 (10)
C2—C3—C4	119.36 (10)	N4—C14—C13	124.22 (13)
C2—C3—H3	119.3 (8)	N4—C14—H14	116.4 (10)
C4—C3—H3	121.3 (8)	C13—C14—H14	119.4 (10)
N2—C4—C3	122.25 (10)	O1—C15—C2	108.29 (9)
N2—C4—C5	115.80 (10)	O1—C15—H15A	109.5 (8)
C3—C4—C5	121.95 (10)	C2—C15—H15A	110.0 (8)
N3—C5—C6	122.61 (10)	O1—C15—H15B	110.2 (8)
N3—C5—C4	116.15 (10)	C2—C15—H15B	111.0 (8)
C6—C5—C4	121.24 (10)	H15A—C15—H15B	107.9 (11)
C7—C6—C5	119.03 (11)	O1—C16—C17	108.01 (10)
C7—C6—H6	122.1 (9)	O1—C16—H16A	109.7 (8)
C5—C6—H6	118.8 (9)	C17—C16—H16A	109.7 (8)
C6—C7—C8	118.99 (11)	O1—C16—H16B	109.5 (9)
C6—C7—H7	120.0 (9)	C17—C16—H16B	110.4 (8)
C8—C7—H7	121.0 (9)	H16A—C16—H16B	109.5 (12)
C9—C8—C7	118.05 (11)	C18—C17—C16	124.63 (15)
C9—C8—H8	121.2 (9)	C18—C17—H17	120.8 (11)
C7—C8—H8	120.7 (9)	C16—C17—H17	114.5 (11)
N3—C9—C8	124.25 (11)	C17—C18—H18A	116.3 (12)
N3—C9—H9	115.5 (9)	C17—C18—H18B	125.0 (12)
C8—C9—H9	120.3 (9)	H18A—C18—H18B	118.4 (16)
N4—C10—C11	122.32 (11)		
			
C1—N1—N2—C4	0.15 (16)	C5—C6—C7—C8	−0.24 (18)
N2—N1—C1—C2	−0.61 (16)	C6—C7—C8—C9	0.57 (18)
N2—N1—C1—C10	178.92 (9)	C5—N3—C9—C8	−0.14 (18)
N1—C1—C2—C3	0.42 (15)	C7—C8—C9—N3	−0.4 (2)
C10—C1—C2—C3	−179.06 (10)	C14—N4—C10—C11	−0.41 (18)
N1—C1—C2—C15	−177.88 (10)	C14—N4—C10—C1	178.51 (11)
C10—C1—C2—C15	2.64 (17)	N1—C1—C10—N4	−164.56 (10)
C1—C2—C3—C4	0.20 (15)	C2—C1—C10—N4	14.96 (16)
C15—C2—C3—C4	178.58 (10)	N1—C1—C10—C11	14.38 (15)
N1—N2—C4—C3	0.49 (16)	C2—C1—C10—C11	−166.10 (11)
N1—N2—C4—C5	−179.18 (9)	N4—C10—C11—C12	0.07 (19)
C2—C3—C4—N2	−0.66 (16)	C1—C10—C11—C12	−178.81 (11)
C2—C3—C4—C5	179.00 (10)	C10—C11—C12—C13	0.1 (2)
C9—N3—C5—C6	0.51 (17)	C11—C12—C13—C14	0.0 (2)
C9—N3—C5—C4	−178.47 (10)	C10—N4—C14—C13	0.6 (2)
N2—C4—C5—N3	−178.91 (10)	C12—C13—C14—N4	−0.4 (2)
C3—C4—C5—N3	1.41 (15)	C16—O1—C15—C2	−173.64 (9)
N2—C4—C5—C6	2.09 (15)	C3—C2—C15—O1	−2.59 (14)
C3—C4—C5—C6	−177.59 (10)	C1—C2—C15—O1	175.66 (9)
N3—C5—C6—C7	−0.32 (18)	C15—O1—C16—C17	−176.11 (10)
C4—C5—C6—C7	178.61 (10)	O1—C16—C17—C18	−126.92 (14)

Symmetry codes: (i) −*x*, −*y*+1, −*z*+1; (ii) −*x*+1, *y*−1/2, −*z*+3/2; (iii) *x*, *y*, *z*+1; (iv) −*x*, *y*+1/2, −*z*+3/2; (v) −*x*+1, −*y*+1, −*z*+1; (vi) −*x*+1, *y*+1/2, −*z*+3/2; (vii) −*x*+1, *y*−1/2, −*z*+1/2; (viii) *x*, −*y*+3/2, *z*+1/2; (ix) −*x*, *y*−1/2, −*z*+3/2; (x) *x*, −*y*+1/2, *z*+1/2; (xi) −*x*, *y*−1/2, −*z*+1/2.

### Hydrogen-bond geometry (Å, º)

*Cg* is the centroid of pyridine ring *B* (N3/C5—C9).

**Table:**

*D*—H···*A*	*D*—H	H···*A*	*D*···*A*	*D*—H···*A*
C8—H8···N1^vi^	0.966 (16)	2.585 (16)	3.4104 (15)	143.4 (12)
C15—H15*B*···*Cg*^v^	0.994 (15)	2.990 (15)	3.8760 (13)	149.0 (11)

Symmetry codes: (v) −*x*+1, −*y*+1, −*z*+1; (vi) −*x*+1, *y*+1/2, −*z*+3/2.
